# Hypermethylation of ribosomal DNA in human breast carcinoma

**DOI:** 10.1054/bjoc.1999.0955

**Published:** 2000-01-18

**Authors:** P S Yan, F J Rodriguez, D E Laux, M R Perry, S B Standiford, T H-M Huang

**Affiliations:** Departments of Pathology and Anatomical Sciences and Surgery, Ellis Fischel Cancer Center, University of Missouri, 115 Business Loop I-70 West, Columbia, MO, 65203, USA

**Keywords:** DNA hypermethylation, ribosomal DNA, breast cancer

## Abstract

We examined the methylation status of the transcribed domain of ribosomal DNA (rDNA) in 58 patients with breast cancer. The mean percent of methylation was significantly higher in breast tumours than that of normal control samples (*P*< 0.0001). This increased rDNA methylation was associated with oestrogen receptor non-expression (*P*< 0.0273) and with moderately or poorly differentiated tumours as compared to well differentiated tumours (*P*< 0.0475). Our results suggest that rDNA can be a useful marker for monitoring aberrant methylation during breast tumour progression. © 2000 Cancer Research Campaign


*British Journal of Cancer* (2000) **82**(3), 514–517
© 2000 Cancer Research Campaign
DOI: 10.1054/ bjoc.1999.0955, available online at http://www.idealibrary.com on IDEAL®

### Hypermethylation of ribosomal DNA in human breast carcinoma

**PS Yan[1], FJ Rodriguez[1], DE Laux[1], MR Perry[1], SB Standiford[2] and TH-M Huang[1]**

Departments of [1]Pathology and Anatomical Sciences and [2]Surgery, Ellis Fischel Cancer Center, University of Missouri, 115 Business Loop I-70 West, Columbia, MO 65203, USA

**Summary** We examined the methylation status of the transcribed domain of ribosomal DNA (rDNA) in 58 patients with breast cancer. The mean percent of methylation was significantly higher in breast tumours than that of normal control samples ($P < 0.0001$). This increased rDNA methylation was associated with oestrogen receptor non-expression ($P < 0.0273$) and with moderately or poorly differentiated tumours as compared to well differentiated tumours ($P < 0.0475$). Our results suggest that rDNA can be a useful marker for monitoring aberrant methylation during breast tumour progression. © 2000 Cancer Research Campaign

**Keywords**: DNA hypermethylation; ribosomal DNA; breast cancer

In human cancer, DNA hypermethylation is known to occur in CpG islands, which are 1- to 2-kb GC-rich regions frequently located within the 5′ ends of about 60% of all genes (Laird and Jaenisch, 1994). This type of epigenetic mutation has been shown to be associated with transcriptional silencing of tumour suppressor genes in neoplasia (Baylin et al, 1997). The abnormal event is generally accepted as a stochastic process in tumour cells with a hypermethylator phenotype (Pfeifer et al, 1990; Jones, 1996; Huang et al, 1999). The random process may occur at CpG sites within the 5′ regulatory regions of critical tumour suppressor genes. The resulting progressive silencing of transcription can provide these cells with a greater proliferative advantage (Jones, 1996). In addition to classical genetic mutations, DNA hypermethylation plays a significant role in promoting tumorigenesis.

Abundant ribosomal DNA shares some characteristics with single-copy CpG islands. The entire 13.3-kb transcriptional domain of ribosomal DNA (rDNA) is GC-rich, but is much longer than a typical 1- to 2-kb CpG island (Worton et al, 1988). In normal human cells, the rDNA transcribed domain is predominately unmethylated, and has been associated with active transcription of 18S, 5.8S and 28S RNA subunits (Dante et al, 1992; Gonzalez et al, 1992). Juxtaposed to the 3′ end of the transcribed domain is a low GC-containing non-transcribed spacer (30-kb) known to contain methylated CpG sites (Brock and Bird, 1997). Approximately 400 copies of rDNA per haploid genome are located on the short arms of human acrocentric chromosomes (Worton et al, 1988). These repeat units are arranged in head-to-tail arrays with each chromosome cluster containing approximately 80 copies (Sakai et al, 1995).

Since both rDNA and CpG islands share similar properties, we sought to determine whether rDNA is subject to aberrant methylation in breast cancer. Methylation analysis was performed by Southern hybridization using the entire transcribed region as a probe in a group of patients with infiltrating ductal carcinomas. The resulting rDNA methylation data were used to examine their association with patients' clinicopathological parameters.

*Received 4 January 1999*
*Revised 12 July 1999*
*Accepted 15 July 1999*

Correspondence to: TH-M Huang

### PATIENTS AND METHODS

### Patients and samples

Breast tumour specimens were obtained from 58 patients undergoing partial or complete mastectomies at the Ellis Fischel Cancer Center (Columbia, MO, USA). Specimen collection and tissue analyses were approved by the Institutional Review Board of the University of Missouri Health Science Center. Clinicopathological parameters and TNM (Tumour-Nodal-Metastasis) classification were performed using standard criteria (Beahrs, 1989). All tumours were classified as infiltrating ductal carcinomas. The oestrogen receptor (ER) and progesterone receptor (PR) status of tumour tissues was determined by either the dextran-coated charcoal assay (negativity defined as ≤ 3 fmol $mg^{-1}$ ligand bound protein) or the immunoperoxidase technique (negativity defined as ≤ 20% of tumour nuclei stained positive). Non-neoplastic breast tissue was also obtained from ten study subjects and used as 'normal controls'. High-molecular-weight DNA was isolated using the QIAamp Tissue Kit™ (Qiagen Inc, Chatsworth, CA, USA).

### Methylation analysis by Southern hybridization

Genomic DNA (approximately 2.5 μg) from breast tissues was digested to completion with methylation-sensitive *Hpa*II (cuts C↓CGG, but not $C^{m}CGG$; m: methylated) or its methylation-insensitive isoschizomer *Msp*I (cuts both C↓CGG and C↓$^{m}$CGG). The restriction products were separated on 1.0% agarose gels and transferred to nylon membranes. The membranes were hybridized with $^{32}$P-labelled pHsrDNA5.1 and pHsrDNA7.9 probes (Figure 1A) at 70°C in 10 ml of High Efficiency Hybridization solution (Molecular Research, Inc., Cincinnati, OH, USA). Probes were radiolabelled using the Multiprime DNA Labelling System

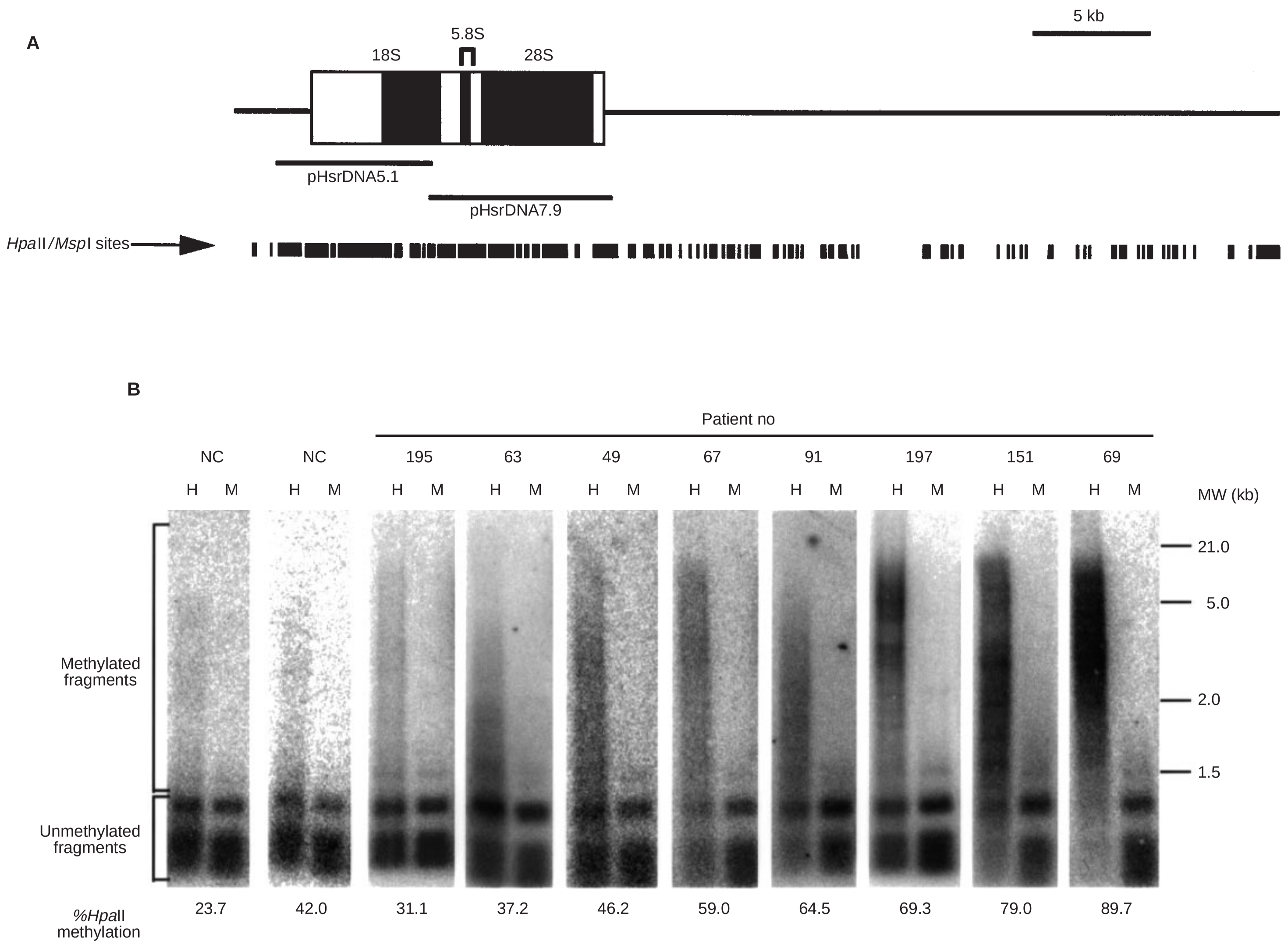

**Figure 1** (**A**) Map of a single ribosomal DNA repeat unit showing the positions of probes (pHsrDNA5.1 and pHsrDNA7.9) used in Southern analysis. The transcriptional units 18S, 5.8S and 28S RNA are shown in filled boxes and the internal and external transcribed spacers are represented in unshaded boxes. Vertical bars mark the relative positions of *Hpa*II/*Msp*I (CCGG) sites in the repeat unit. (**B**) Methylation analysis of ribosomal DNA in primary breast tumours and normal breast tissues (NC). Genomic DNA was digested with methylation-sensitive *Hpa*II (H) or its methylation-insensitive isoschizomer *Msp*I (M), and subjected to Southern hybridization using the combined pHsrDNA5.1 and pHsrDNA7.9 as probes. The methylated and unmethylated fragments are indicated at left and molecular weight markers are shown at right. Eight patient tumour samples with increasing % methylation were selected and shown with their respective densitometric data

© 2000 Cancer Research Campaign

**Table 1** Clinicopathological features of patients with infiltrating ductal carcinoma of the breast.

| **Patient characteristics** | ***n*** | **Age (mean ± SD)** | **%rDNA methylation (mean ± SD)** | ***P*-value[a]** |
|---|---|---|---|---|
| Normal vs. tumour | | | | |
| Normal breast tissue | 10 | 57.9 ± 15.2 | 41.0 ± 10.2 | **0.0001** |
| Tumour breast tissue | 58 | 58.4 ± 15.7 | 62.4 ± 14.4 | |
| Age at diagnosis (years) | | | | |
| <50 | 20 | 41.8 ± 4.5 | 67.1 ± 13.5 | NS (0.0743) |
| ≥50 | 38 | 67.2 ± 11.8 | 60.0 ± 14.4 | |
| Oestrogen receptor (ER) | | | | |
| Positive | 31 | 64.1 ± 15.7 | 58.5 ± 16.0 | **0.0273** |
| Negative | 27 | 51.8 ± 13.0 | 67.0 ± 10.8 | |
| Progesterone receptor (PR) | | | | |
| Positive | 20 | 63.8 ± 16.4 | 59.5 ± 14.9 | NS (0.1430) |
| Negative | 34 | 54.9 ± 14.3 | 65.5 ± 13.8 | |
| Combined ER/PR status | | | | |
| ER+/PR+ | 22 | 64.4 ± 16.5 | 59.0 ± 15.1 | NS (0.0848) |
| ER+/PR– | 9 | 63.4 ± 14.4 | 57.2 ± 19.0 | |
| ER–/PR– | 26 | 51.9 ± 13.3 | 66.9 ± 10.0 | |
| Mitotic frequency | | | | |
| <20 HPF[b] | 37 | 60.7 ± 16.6 | 61.4 ± 13.8 | NS (0.1104) |
| ≥20 HPF | 15 | 52.0 ± 13.8 | 68.2 ± 13.6 | |
| Tumour differentiation | | | | |
| In all the tumor tissues | | | | |
| WD[c] | 3 | 76.0 ± 17.3 | 49.2 ± 18.3 | **0.0475** |
| MD/PD | 50 | 56.8 ± 15.0 | 64.8 ± 12.7 | |
| In ER+/PR+ subgroup | | | | |
| WD/MD | 16 | 65.8 ± 16.1 | 57.4 ± 13.8 | **0.0376** |
| PD | 4 | 59.5 ± 17.9 | 73.8 ± 8.70 | |
| In ER–/PR– subgroup | | | | |
| MD | 12 | 54.0 ± 14.7 | 68.2 ± 7.1 | NS (0.5828) |
| PD | 14 | 50.1 ± 12.2 | 65.7 ± 13.6 | |
| TNM classification[d] | | | | |
| I | 9 | 63.4 ± 14.6 | 62.6 ± 14.7 | NS (0.4966) |
| II | 28 | 58.6 ± 15.3 | 63.7 ± 13.5 | |
| III | 7 | 52.0 ± 10.9 | 61.5 ± 16.5 | |
| IV | 5 | 51.6 ± 18.7 | 73.2 ± 9.30 | |

[a]Statistical analyses performed on the rDNA methylation values vs. patient characteristics using PROC TTEST or PROC GLM (SAS System, Cary, NC). Statistically significant ($P < 0.05$). NS, not significant. [b]HPF, high power field, 40×. [c]WD, well differentiated; MD, moderately differentiated; PD, poorly differentiated. All the WD were in the ER+/PR+ subgroup. [d]Classification according to the TNM system (Beahrs, 1989).

(Amersham-Pharmacia Biotech, Piscataway, NJ, USA). Washing was performed once for 20 min in 0.1% sodium dodecyl sulphate (SDS)–0.5× SSC (1× SSC is 0.15 M sodium chloride and 0.015 M sodium citrate, pH 7.0) and thrice for 20 min each in 0.1% SDS–0.2× SSC at 70°C. The hybridized membranes were subjected to image analysis with a Molecular Dynamics PhosphorImager (Molecular Dynamics, Sunnyvale, CA, USA). Band intensities were quantified using the densitometric function of the ImageQuant software (Molecular Dynamics). Methylation was expressed as the percentage of the intensity of the methylated fragments to the combined intensities of all the fragments in *Hpa*II sample lanes as depicted in Figure 1B.

### Statistical analyses

The percent rDNA methylation values and the patient ages were reported as mean ± standard deviation (s.d.). The association between percent rDNA hypermethylation data and patients' clinicopathological data were analysed using the SAS procedure PROC TTEST for comparisons having two variables and PROC GLM for comparisons having three or more variables (Release 6.12, SAS Institute, Cary, NC, USA). Statistical significance was established as $P < 0.05$.

### RESULTS

Methylation analysis was conducted by Southern hybridization in 58 primary breast tumours and ten normal controls using probes spanning the transcribed domain of rDNA (Figure 1A). A total of 280 *Hpa*II/*Msp*I sites located within this region were examined. Representative results are shown in Figure 1B. In control samples, the pattern (fragments with size < 1 kb) of methylation-sensitive *Hpa*II restriction was largely the same as that of methylation-insensitive *Msp*I restriction, indicating that the majority of these sites within the transcribed domain were unmethylated. Some fainter fragments appeared as smears (> 1 kb) in the *Hpa*II-restricted lanes, suggesting a minor proportion of these sites were methylated and protected from restriction, consistent with a previous observation in normal cells (Brock and Bird, 1997). In tumour samples, the patterns between methylation-sensitive and -insensitive restrictions were often different. The predominant fragments of the *Hpa*II-restricted fragments shifted into regions of higher molecular weights (> 1 kb); a smear of varying length and band intensity was seen in these regions due to the differing degrees of methylation in tumour samples. The *Msp*I-digested fragments in tumours remained essentially similar to those of the normal control samples.

 *© 2000 Cancer Research Campaign*

The resulting methylation data and patients' clinicopathological features are summarized in Table 1. An overall increase of rDNA methylation was seen in ~80% of the breast tumours examined. The mean percent methylation of breast tumours was significantly higher than that of normal breast tissue samples ($P < 0.0001$). The percent rDNA methylation was also significantly higher in ER-negative tumours than in ER-positive tumours ($P < 0.0273$), and in moderately or poorly differentiated tumours in comparison to well-differentiated tumours ($P < 0.0475$). As we further subdivided groups by ER and PR combined expression, the poorly differentiated tumours had significantly higher rDNA methylation than the moderately or well differentiated tumours within the ER-positive and PR-positive subgroup ($P < 0.0376$). rDNA methylation was essentially the same between moderately and poorly differentiated tumours in the ER-negative and PR-negative subgroup ($P < 0.5828$; none of the well differentiated tumours belonged to this subgroup). When the diagnosed age of 50 years old was used as the cutoff, tumours from younger patients (≤ 50 years old) had higher rDNA methylation though the observed differences were of borderline statistical significance (*P*-value of 0.0743). Interestingly, this inverse association between age at diagnosis and rDNA methylation was observed in most of the tumour subgroups as shown in the 'Age' and '% rDNA methylation' columns in Table 1. The percent rDNA methylation was not associated with tumour TNM classifications, PR negativity, or tumour mitotic frequency.

### DISCUSSION

This study presents evidence that, in addition to single-copy CpG islands, abundant rDNA sequences are another type of methylation substrate in breast cancer. Our data indicated that increased rDNA methylation was often found in subgroups of patients with tumour undifferentiation and with younger diagnosed age (≤ 50 years old). Interestingly, rDNA hypermethylation was also correlated with ER negativity ($P < 0.0273$). Previous studies have shown that hypermethylation of the *ER* CpG island was associated with the lack of ER expression in ER-negative breast cancer cells (Ottaviano et al, 1994) and in 25% of ER-negative tumours (Lapidus et al, 1996). These studies also indicated that methylation silencing of the *ER* gene might be a primary cause responsible for some breast cancer patients subsequently becoming insensitive to anti-oestrogen therapies. Together with these previous results, we hypothesize that hypermethylation of rDNA and the *ER* CpG island can be a related event in breast cancer. If this assumption were further proven, then the finding could support a generalized mechanism governing the epigenetic event. Since DNA hypermethylation may arise as a stochastic process in tumour cells as indicated earlier, abundant copies of rDNA can be more available than single-copy CpG islands in this chance event.

We, therefore, reason that rDNA is a potential marker for tumours having a greater propensity to methylate their genome (i.e. hypermethylator phenotype). In the aforementioned clinical correlation, the random methylation process can simultaneously occur in both rDNA and the *ER* CpG island in this subgroup of breast tumours. In other breast tumours with a hypermethylator phenotype, aberrant methylation may have already occurred in abundant rDNA, but not yet in the *ER* CpG island. Thus, can the status of hypermethylated rDNA predict this type of tumour having a high likelihood of developing an ER-negative phenotype due to subsequent hypermethylation of the *ER* CpG island? Future methylation studies of rDNA together with *ER* and other gene CpG islands in breast cancer are needed to address this question.

In conclusion, we have shown for the first time that rDNA hypermethylation occurs in breast tumours, and may be an important marker for this epigenetic event in neoplasia. Our finding highlights the need for further investigations of rDNA hypermethylation and its relationship to the development of breast carcinoma.

### ACKNOWLEDGEMENTS

The authors wish to thank Professor Adrian Bird at the University of Edinburgh for providing plasmids pHsrDNA5.1 and pHsrDNA7.9. This work was supported by National Cancer Institute grant CA-69065 (TH-MH) and by US Army Medical Research Command grant DAMD17-98-1-8214 (TH-MH).

### REFERENCES

Baylin SB, Herman JG, Graff JR, Vertino PM and Issa JP (1997) Alterations in DNA methylation: a fundamental aspect of neoplasia. In: *Advances in Cancer Research*, Vol. 72, Vande Woude GF and Klein G (eds) pp. 141–196. Academic Press: San Diego

Beahrs OH (1989) *American Joint Committee on Cancer: Manual for Staging of Cancer*, pp. 93–99. Lippincott: Philadelphia

Brock GJR and Bird A (1997) Mosaic methylation of the repeat unit of the human ribosomal RNA genes. *Hum Mol Genet* **6:** 451–456

Dante R, Percy ME, Baldini A, Markovic VD, Miller DA, Rocchi M, Niveleau A and Miller OJ (1992) Methylation of the 5′ end flanking sequences of the ribosomal DNA in human and a human–hamster cell line. *J Cell Biochem* **50:** 357–362

Gonzalez IL, Wu S, Li WM, Kuo BA and Sylvester EJ (1992) Human ribosomal RNA intergenic spacer sequence. *Nucleic Acids Res* **20:** 5846–5847

Huang TH-M, Perry MR and Laux DE (1999) Methylation profiling of CpG islands in human breast cancer cells. *Hum Mol Genet* **8:** 459–470

Jones PA (1996) DNA methylation errors and cancers. *Cancer Res* **56:** 2463–2467

Laird PW and Jaenisch R (1994) DNA methylation and cancer. *Hum Mol Genet* **3:** 1487–1495

Lapidus RG, Ferguson AT, Ottaviano YL, Parl FF, Smith HS, Weitzman SA, Baylin SB, Issa J-P and Davidson NE (1996) Methylation of estrogen and progesterone receptor gene 5′ CpG islands correlates with lack of oestrogen and progesterone receptor gene expression in breast tumours. *Clin Cancer Res* **2:** 805–810

Ottaviano YL, Issa JP, Parl FF, Smith HS, Baylin SB and Davidson NE (1994) Methylation of the estrogen receptor gene CpG island marks loss of estrogen receptor expression in human breast cancer cells. *Cancer Res* **54:** 2552–2555

Pfeifer GP, Steigerwald SD, Hansen RS, Gartler SM and Riggs AD (1990) Polymerase chain reaction-aided genomic sequencing of an X chromosome-linked CpG island: methylation patterns suggest clonal inheritance, CpG site autonomy, and an explanation of activity state stability. *Proc Natl Acad Sci USA* **87:** 8252–8256

Sakai K, Ohta T, Minoshima S, Kudoh J, Wang Y, de Jong PJ and Shimizu N (1995) Human ribosomal RNA gene cluster: identification of the proximal end containing a novel tandem repeat sequence. *Genomics* **26:** 521–526

Worton RG, Sutherland J, Sylvester JE, Willard HF, Bodrug S, Dube I, Duff C, Kean V, Ray PN and Schmickel RD (1988) Human ribosomal RNA genes: orientation of the tandem array and conservation of the 5′ end. *Science* **239:** 64–68

© 2000 Cancer Research Campaign
